# The Influence of Special Environments on SiC MOSFETs

**DOI:** 10.3390/ma16186193

**Published:** 2023-09-13

**Authors:** Zhigang Li, Jie Jiang, Zhiyuan He, Shengdong Hu, Yijun Shi, Zhenbo Zhao, Yigang He, Yiqiang Chen, Guoguang Lu

**Affiliations:** 1 School of Electrical Engineering and Automation, Hefei University of Technology, Hefei 230009, China; leiyu27@126.com (Z.L.);; 2 School of Microelectronics and Communication Engineering, Chongqing University, Chongqing 400044, China; 3 The Science and Technology on Reliability Physics and Application of Electronic Component Laboratory, China Electronic, Product Reliability and Environmental Testing Research Institute, Guangzhou 510610, China

**Keywords:** SiC MOSFETs, hydrogen gas, HAST, planar gate structure, dual gate groove structure, asymmetric groove structure

## Abstract

In this work, the influences of special environments (hydrogen gas and high temperature, high humidity environments) on the performance of three types of SiC MOSFETs are investigated. The results reveal several noteworthy observations. Firstly, after 500 h in a hydrogen gas environment, all the SiC MOSFETs exhibited a negative drift in threshold voltage, accompanied by an increase in maximum transconductance and drain current (@ V_GS_/V_DS_ = 13 V/3 V). This phenomenon can be attributed to that the hydrogen atoms can increase the positive fixed charges in the oxide and increase the electron mobility in the channel. In addition, high temperature did not intensify the impact of hydrogen on the devices and electron mobility. Instead, prolonged exposure to high temperatures may induce stress on the SiO_2_/SiC interface, leading to a decrease in electron mobility, subsequently reducing the transconductance and drain current (@ V_GS_/V_DS_ = 13 V/3 V). The high temperature, high humidity environment can cause a certain negative drift in the devices’ threshold voltage. With the increasing duration of the experiment, the maximum transconductance and drain current (@ V_GS_/V_DS_ = 18V (20 V)/3 V) gradually decreased. This may be because the presence of moisture can lead to corrosion of the devices’ metal contacts and interconnects, which can increase the devices’ resistance and lead to a decrease in the devices’ maximum transconductance and drain current.

## 1. Introduction

Power electronics play a crucial role in various applications, including electric vehicles, renewable energy systems, and industrial automation [1,2,3,4,5]. As the demand for higher efficiency and power density increases, silicon-based power devices face limitations due to their inherent material properties. To address these limitations, alternative power semiconductor materials such as silicon carbide (SiC) have been developed [6,7,8,9]. SiC-based metal oxide semiconductor field-effect transistors (MOSFETs) have garnered significant attention in recent years, due to their higher breakdown voltage, lower on-resistance, and faster switching speed [6,7,8,9,10]. These characteristics make them ideal for high power and high frequency applications, where energy efficiency and compactness are critical. Apart from their excellent electrical properties, SiC MOSFETs also have superior thermal conductivity compared to silicon devices [11,12,13], which enables them to handle higher current densities and reduces the need for extensive cooling systems. Furthermore, SiC MOSFETs offer enhanced ruggedness and reliability, making them well-suited for harsh operating conditions [14,15,16,17]. They exhibit excellent resistance to high voltages, high temperatures, and high frequency switching, which are common challenges in power electronics applications. This reliability advantage reduces maintenance requirements and extends the lifespan of the devices, resulting in improved system availability and reduced downtime.

However, the performance of SiC MOSFETs in special environments, such as the presence of hydrogen atoms and high temperature, high humidity environments, needs to be thoroughly investigated. Hydrogen ion implantation is often used to alter the SiC material’s properties and improve the electrical performance of SiC-based devices, such as enhancing the electron mobility in SiC MOSFETs [18,19,20]. This increase in mobility can lead to an improved drain current, resulting in a lower on-resistance and a reduced power loss for SiC MOSFETs. The packaging materials used for SiC MOSFETs often contain hydrogen atoms that can be released over time. When the hydrogen atoms are released from the packaging materials [21,22,23], they can be diffused into the SiC material, which will affect the devices’ electrical properties, leading to a drift in the device parameters. As mentioned in [22], the hydrogen atoms should be maintained at concentrations below 10 ppb (parts per billion); however, the concentration of hydrogen atoms released from the packaging material may be close to 2500 ppb. In addition, SiC is also highly favored in hydrogen sensor research [24,25,26]. To fully understand the impact of hydrogen atoms on SiC MOSFETs, comprehensive studies are needed. Experimental investigations can provide valuable insights into the behavior of hydrogen atoms in SiC MOSFETs and their effects on device performance.

Similarly, high temperature, high humidity environments are commonly encountered in industrial applications, such as power plants and automotive systems [27,28,29,30]. One of the primary concerns in high temperature and high humidity environments is the potential for moisture absorption [27,28,29,30]. Moisture can permeate the package, leading to the formation of water droplets or condensation on the device surface. The presence of moisture can lead to corrosion of the devices’ metal contacts and interconnects, which can increase the resistance of these components, leading to a higher power losses and reduced device efficiency. The combination of high temperature and humidity can speedily lead to the formation of moisture and water vapor on the device surface, potentially causing corrosion. These environmental factors can significantly impact the performance of SiC MOSFETs, necessitating a comprehensive understanding of their behavior in such conditions. Although there have been studies on SiC MOSFETs in high temperature, high humidity, and other environments [31,32,33,34], there has been no comparative study of several different structures of SiC MOSFETs, and it is impossible to determine which structure of devices is more suitable for working in such high temperature, high humidity environments. There is an urgent need to carry out relevant research.

Therefore, this study aims to investigate the performance of three types of SiC MOSFETs in hydrogen gas and high temperature, high humidity environments. The results will contribute to the understanding of SiC MOSFETs’ suitability and reliability in these special environments and provide insights into future possible applications. In the following sections, the experimental setup in this study will be described. The results and discussions will then be presented, followed by the conclusions.

## 2. Experimental Setup

**Figure 1:**
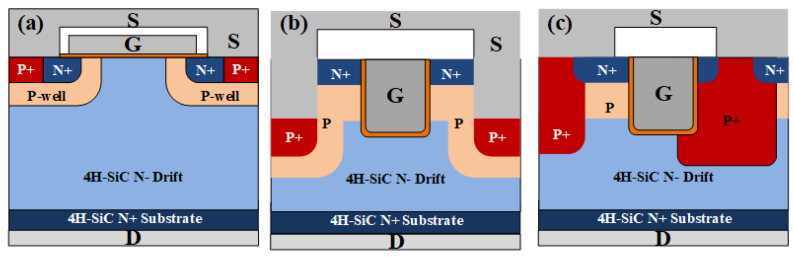
Schematic diagram of SiC VDMOS with (**a**) planar gate structure, (**b**) double trench structure, and (**c**) asymmetric trench structure.

**Figure 2:**
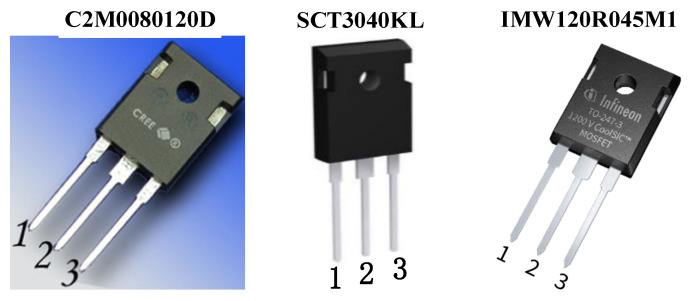
Package schematic of the three types of SiC MOSFETs. Note: 1, 2, and 3 are the gate, source, and drain electrodes of the device, respectively.

The SiC MOSFETs used in this study include the planar gate structure [35], dual trench structure [36,37] and asymmetric trench structure [38,39,40,41,42,43] (Figure 1). The SiC MOSFET with a planar gate structure (Figure 1a) has the gate electrode placed on top of the gate insulator, which is typically made of silicon dioxide (SiO_2_) or a high-k dielectric. The gate insulator separates the gate electrode from the channel region, controlling the flow of current. This structure allows for simple fabrication and provides good control over the channel conductivity. The SiC MOSFET with a dual trench gate structure (Figure 1b) features a trench etched into the SiC substrate, with the gate electrode positioned inside the trench. The gate insulator is formed on the trench sidewalls, isolating the gate electrode from the channel region. This structure increases the gate channel capacitance and reduces the on-resistance of the device. It also provides better control over the electric field distribution, improving the overall performance of the MOSFET. The SiC MOSFET with an asymmetric trench gate structure (Figure 1c) has a trench etched deeper on one side than on the other, creating an asymmetric electric field distribution and allowing for better control over the device characteristics. The gate insulator is formed on the trench sidewalls, as in the symmetric trench gate structure. The asymmetric trench gate structure can provide improved performance in terms of on-resistance, breakdown voltage, and switching speed. Overall, the planar gate structure is simpler to fabricate and offers good control over the channel conductivity. The symmetric and asymmetric trench gate structures provide enhanced performance in terms of on-resistance, breakdown voltage, and switching speed, but they require more complex fabrication processes. For this research, the SiC MOSFET devices used are the C2M0080120D (planar gate structure) from Cree, SCT3040KL (dual trench gate structure) from Rohm, and IMW120R045M1 (asymmetric trench gate structure) from Infineon. Their rated current/voltage values are 31.6A/1200 V, 55A/1200 V, and 52A/1200 V, respectively. All three devices employ the TO-247 package (Figure 2). The test settings are provided in Table 1. These details outline the configuration used for testing in this study.

**Figure 3:**
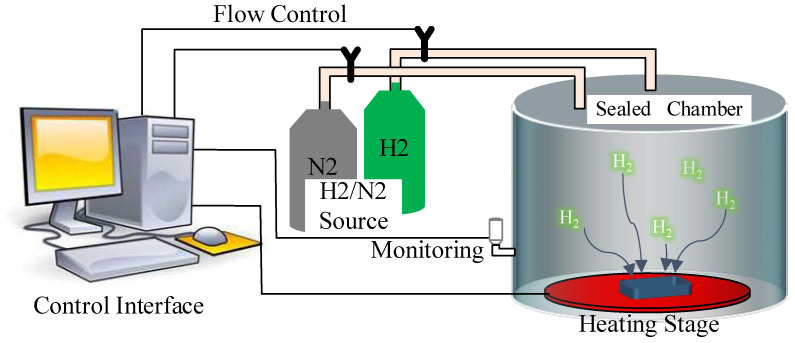
Atmospheric environment simulation test system.

Regarding the hydrogen test setup, in this study, a self-developed atmosphere simulation test system was used to conduct hydrogen effect experiments on the three types of SiC MOSFETs, as shown in Figure 3. The atmosphere simulation test system mainly consists of a gas source, control interface, sealed chamber to conduct the hydrogen effect experiments, component monitor, and heating stage. By inputting the type and concentration ratio of the required gas in the control interface, the corresponding atmosphere can be obtained in the sealed chamber. Considering the flammable and explosive nature of hydrogen, safety precautions were taken by setting the hydrogen content at 4%, with the remaining 96% consisting of nitrogen. During the experiment, the three types of SiC MOSFETs were first placed in the above-mentioned gas environment at room temperature for 500 h, allowing sufficient time for hydrogen to enter the device. Subsequently, the MOSFETs were subjected to the same gas environment at 100 °C for either 200 h or 500 h, which was to observe if the high temperature would accelerate the change in device performance. Following this, the devices were returned to room temperature and kept for an additional 1250 h to determine if the device performance would recover. Finally, the devices were placed at 200 °C for 500 h. Before and after each experiment, the device characteristics of the three types of SiC MOSFETs were characterized using the semiconductor parameter analysis systems (Keysight B1505 and Keysight B1500) to observe the changes in device electrical performance. All the terminals of the SiC power MOSFETs were floating during the hydrogen effect experiments. And due to the strong penetration of hydrogen gas, the devices were not unpacked during the hydrogen effect experiments.

Regarding the high accelerated temperature and humidity stress test (HAST) setup, in this study, the HAST method was employed to investigate the influence of high temperature, high humidity environments on the three types of SiC MOSFETs. HAST tests can increase the pressure of water vapor within the container, which can enable temperatures to exceed 100 °C and accelerate the aging effect of water vapor penetration into the components. An EHS-211MD HAST chamber was used to perform the HAST tests on the three types of SiC MOSFETs. In order to observe the direct impact of the temperature and humidity environmental stress on the SiC MOSFETs and to avoid degradation failure caused by voltage bias, the unbiased HAST (UHAST) test was conducted. Unlike traditional HAST tests, which involve applying voltage bias, the UHAST test eliminates voltage bias to focus solely on the effects of temperature and humidity. The temperature stress, humidity stress, and pressure applied in the chamber during the experiment were set to 130 °C, 85% RH, and 230 kPa. Before and after the HAST test, the characteristics of the three different structures of SiC MOSFETs were primarily characterized every 100 h to observe any changes in device electrical performance. And during the UHAST test, the devices were unpacked.

## 3. Results and Discussion

### 3.1. The Influence of Hydrogen Gas on SiC MOSFETs

**Figure 4:**
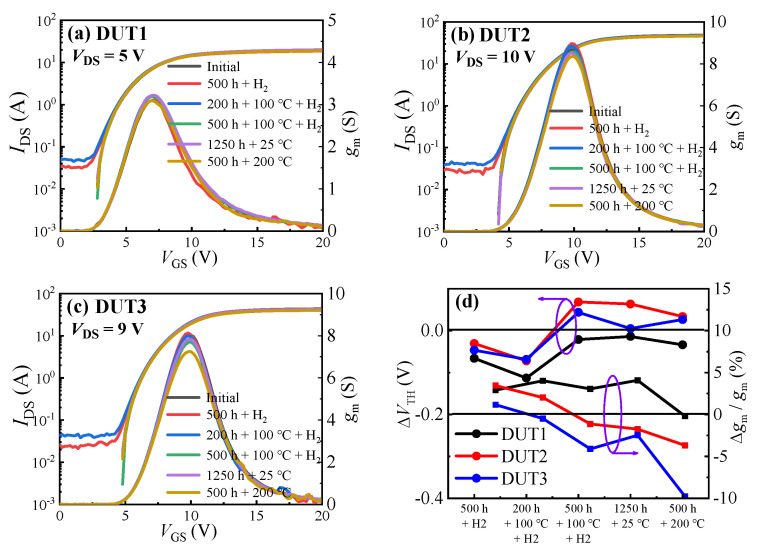
Transfer characteristic curves before and after the hydrogen effect experiments of (**a**) DUT1, (**b**) DUT2, and (**c**) DUT3. (**d**) Summary of the changes in the devices’ *g*_m_max_ and *V*_TH_.

Figure 4 depicts the transfer characteristic curves before and after the hydrogen effect experiments for the three types of SiC MOSFETs. It can be observed that, after 500 h in a hydrogen environment, the planar gate structure SiC MOSFET showed a 2.89% increase in maximum transconductance (*g*_m_max_) and a 0.07 V negative drift in threshold voltage. The dual gate groove structure SiC MOSFET exhibited a 3.34% increase in *g*_m_max_ and a 0.03 V negative drift in threshold voltage. The asymmetric groove structure SiC MOSFET demonstrated a 1.16% increase in *g*_m_max_ and a 0.05 V negative drift in threshold voltage. This implies that all three types of SiC MOSFETs experienced an increase in *g*_m_max_ and a negative drift in threshold voltage after 500 h in a hydrogen environment. Notably, the planar gate structure SiC MOSFET exhibited the largest negative drift in threshold voltage, while the dual gate groove structure SiC MOSFET demonstrated the largest change in *g*_m_max_. As mentioned in [18,19,20,21,44], hydrogen molecules can dissociate into hydrogen atoms under Au catalysis. Then, the hydrogen atoms can lose an electron due to the attraction of the Si nucleus, eventually forming H+ ions. The H+ ions can increase the positive fixed charges in the oxide of SiC MOSFETs, leading to a negative drift in threshold voltage. Additionally, hydrogen atoms can increase the electron mobility in the gate channel of SiC MOSFETs, thereby increasing the devices’ transconductance [18,19,20,21]. According to the following Formula (1) [45], the maximum transconductance of the device is mainly related to the carrier mobility. Therefore, the change in *g*_m_max_ can reflect the change in carrier mobility.

(1) $$g_{m\_\max}=1.3\frac{W_{g}}{L_{g}}C_{\mathrm{ox}}\mu V_{DS}$$

where *W*_g_ and *L*_g_ are the gate width and gate length, respectively, *C*_ox_ is the gate oxide layer capacitance, and *V*_DS_ is the drain voltage. After maximum transconductance, the main reason for the decrease in the transconductance of the device is that the carrier concentration increases with the increase in gate voltage, and the enhanced collision scattering between carriers leads to a decrease in carrier mobility, resulting in a decrease in transconductance.

After the three types of SiC MOSFETs were subjected to a hydrogen environment at 100 °C for 200 h, the devices’ threshold voltage continued to drift negatively, but, except for the planar gate structure SiC MOSFET, the maximum transconductance of the other two structures of SiC MOSFETs started to decrease. The threshold voltage of the three structures of SiC MOSFETs drifted negatively by 0.05 V, 0.04 V, and 0.02 V, respectively. The maximum transconductance of the planar gate structure SiC MOSFET increased by 1.13%, while the maximum transconductance of the other two structures of SiC MOSFETs decreased by 1.43% and 1.66%, respectively. Subsequently, when the three types of SiC MOSFETs were subjected to a 100 °C hydrogen environment for an additional 300 h, notable changes occurred. All the devices exhibited positive drifts in threshold voltage, with values of 0.09 V, 0.14 V, and 0.11 V for the three structures, respectively. Furthermore, the maximum transconductance of all three structures decreased by 0.96%, 3.15%, and 3.61%, respectively. In other words, high temperature does not accelerate the impact of hydrogen on SiC MOSFETs; instead, it leads to device performance degradation. In addition, prolonged exposure to high temperatures may increase the stress on the devices’ SiO_2_/SiC interface, leading to a decrease in electron mobility and subsequently reducing the device transconductance.

To validate this hypothesis, we first placed three types of SiC MOSFETs at room temperature for 1250 h, and then placed them in a 200 °C environment for 500 h to observe the changes in device performance. After being placed at room temperature for 1250 h, the performance of the devices remained largely unchanged. However, after being placed in a 200 °C environment for 500 h, there was no significant change in the threshold voltage of the devices, but their maximum transconductance showed a significant decrease. The maximum transconductance of the three structures of SiC MOSFETs decreased by 4.28%, 1.93%, and 7.30%, respectively. This indicates that high temperature does not increase or decrease the positive fixed charges in the oxide of SiC MOSFETs, and thus does not alter the devices’ threshold voltage. However, prolonged thermal stress increases the stress on the SiO_2_/SiC interface [46], leading to a decrease in electron mobility at the channel and further reducing the devices’ transconductance.

**Figure 5:**
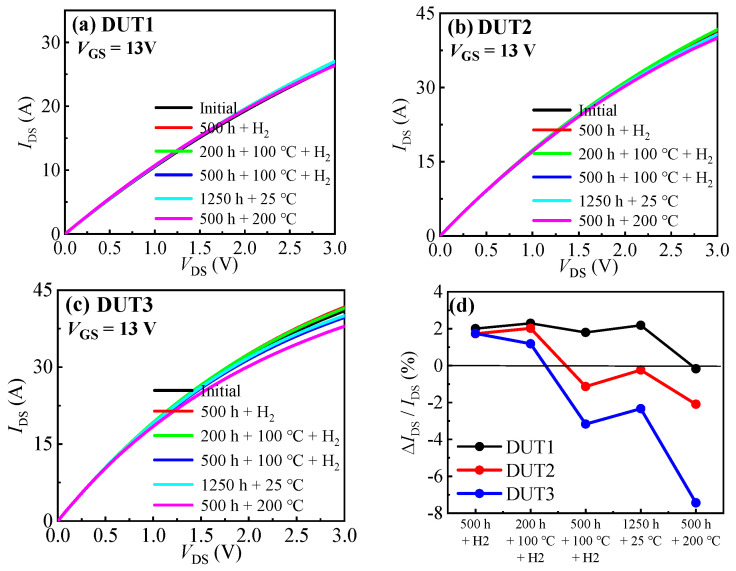
Output characteristic curves before and after the hydrogen effect experiments of (**a**) DUT1, (**b**) DUT2, and (**c**) DUT3. (**d**) Summary of the change in the devices’ *I*_DS_.

Figure 5 illustrates the output characteristic curves of the three types of SiC MOSFETs before and after the experiment. Firstly, after subjecting the devices to 500 h in a hydrogen environment, the conduction currents (@ V_GS_/V_DS_ = 13 V/3 V) of the three types of SiC MOSFETs increased by 2.001%, 1.73%, and 1.75% respectively. The increase in the conduction current of the devices is mainly attributed to the decrease in device threshold voltage and the increase in transconductance. And after 200 h in a 100 °C hydrogen environment, the conduction currents of the devices remained largely unchanged. However, further increasing the storage time in a 100 °C hydrogen environment will result in a significant decrease in the conduction current of the devices. After 500 h in a 100 °C hydrogen environment, the three types of SiC MOSFETs decreased by 0.50%, 3.16%, and 4.35%, respectively. This is due to the increase in device threshold voltage and the decrease in transconductance. As mentioned above, the prolonged exposure to high temperatures may increase the stress on the gate oxide interface of the chip, subsequently leading to a decrease in electron mobility at the channel and reducing the device transconductance. Additionally, after being placed at room temperature for 1250 h, the devices exhibited relatively unchanged performance. However, upon exposure to a 200 °C environment for 500 h, a significant decrease in conduction current was observed. Specifically, the conduction currents of the three SiC MOSFET structures decreased by 2.36%, 1.86%, and 5.10%, respectively. The conduction current fluctuation of asymmetric gate SiC MOSFET was larger than that of planar gate SiC MOSFET. This may result from the fact that the current fabrication process for the planar gate SiC MOSFET is more mature than that for the asymmetric gate SiC MOSFET, resulting in a relatively smaller potential reliability problem for the planar gate SiC MOSFET. On the other hand, the asymmetric gate SiC MOSFET may face greater reliability challenges. Consequently, the performance of the asymmetric gate SiC MOSFET may experience more noticeable variations when exposed to external environmental factors.

**Figure 6:**
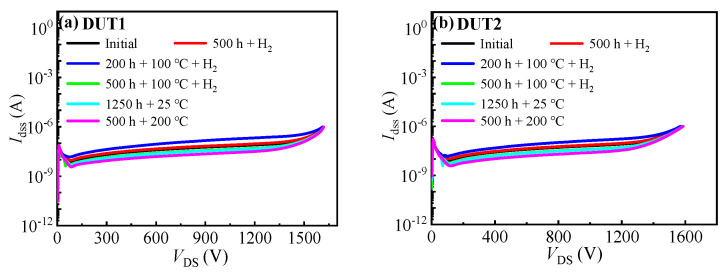

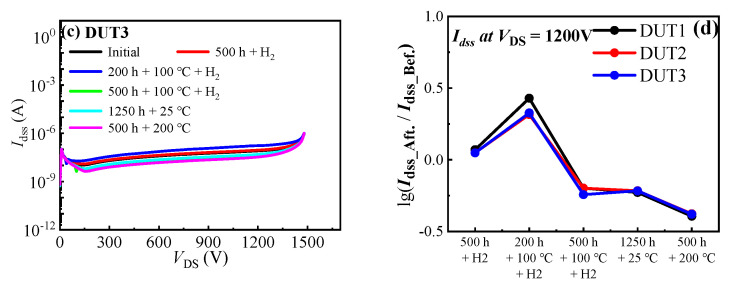
Leakage current characteristic curves before and after the hydrogen effect experiments of (**a**) DUT1, (**b**) DUT2, and (**c**) DUT3. (**d**) Summary of the change in the devices’ *I*_dss_.

Figure 6 depicts the drain leakage current characteristics curves of the three types of SiC MOSFETs before and after the hydrogen effect experiments. Firstly, after subjecting the devices to 500 h in a hydrogen environment, the drain leakage current of all the SiC MOSFETs slightly increased. It is possible that the increase in the devices’ drain leakage current is due to the penetration of hydrogen atoms into the silicon carbide material and their interaction with impurities or defects present in the silicon carbide material. This may lead to the formation of or increase in leakage paths, resulting in an increase in the leakage current of the devices. And after 200 h in a 100 °C hydrogen environment, the drain leakage currents of the three types of SiC MOSFETs continued to increase. However, further increasing the storage time in a 100 °C hydrogen environment will result in a significant decrease in the leakage current of the devices, which may result from the fact that the high temperatures can repair some lattice damage and reduce the defect density in the silicon carbide material, thereby reducing leakage pathways and further decreasing leakage current. This can be validated by observing a significant decrease in leakage current of the device after it is subjected to a high temperature environment of 200 °C for 500 h.

### 3.2. The Influence of High Temperature, High Humidity Environments on SiC MOSFETs

**Figure 7:**
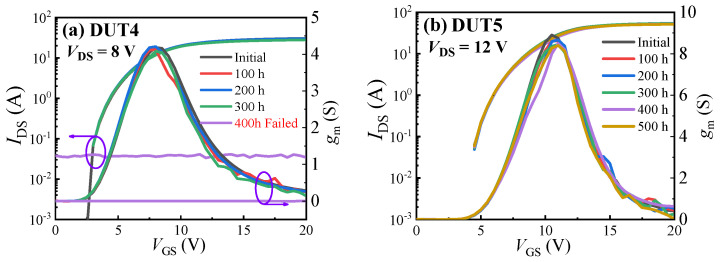

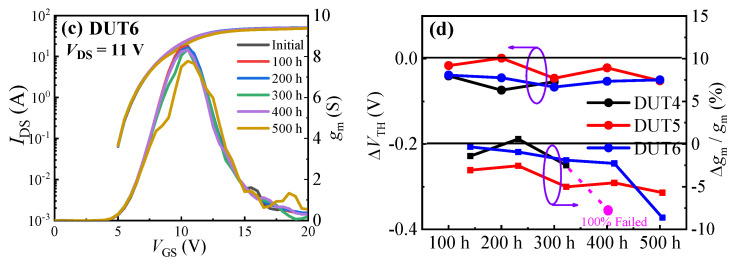
Transfer characteristic curves before and after UHAST of (**a**) DUT4, (**b**) DUT5, and (**c**) DUT6. (**d**) Summary of the change in the devices’ *g*_m_ and *V*_TH_.

**Figure 8:**
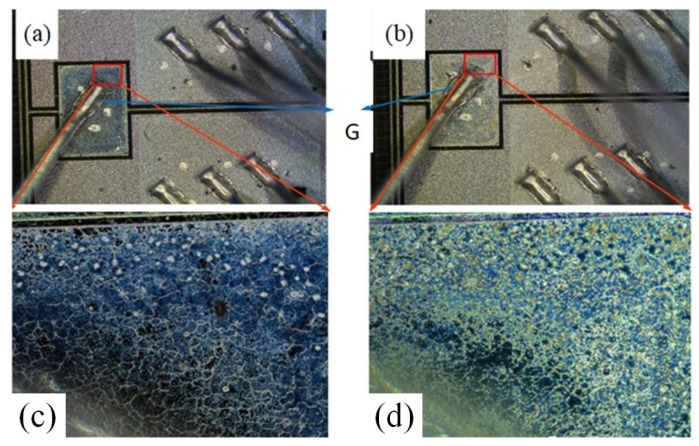
The optical images of the SiC MOSFET (C2M0080120D) before (**a**,**c**) and after (**b**,**d**) UHAST. (**c**,**d**) are partially enlarged images of gate region metal.

Figure 7 displays the transfer characteristics curves of the three types of SiC MOSFETs before and after the UHAST experiments. After subjecting the devices to 400 h of UHAST experiments, the planar gate structure SiC MOSFET failed. Before the failure occurred, the threshold voltage of the device experienced a certain amount of negative drift. No obvious pattern was observed in the variation in the devices’ maximum transconductance. Both the dual gate groove structure and asymmetric groove structure SiC MOSFETs also exhibited a slight negative drift in device threshold voltage and a slight decrease in device maximum transconductance after 500 h of UHAST experiments. The drift amount of threshold voltage did not increase with the duration of the UHAST experiment, but the devices’ maximum transconductances gradually decreased with the duration of the experiment. This may because the presence of moisture in UHAST experiments can lead to corrosion of the devices’ metal contacts and interconnects, which can increase the devices’ resistance and lead to the decrease in the devices’ maximum transconductance. The failure of the planar gate structure SiC MOSFET after 400 h of UHAST experiments could be due to a break in the drain or source electrode of the device. Figure 8 gives the optical images of the C2M0080120D before and after UHAST. The gate metal of SiC MOSFETs is generally copper or aluminum, which are more susceptible to oxidation and corrosion, subsequently causing a disconnection between the gate metal and the bonding wire. The gate metal turned green, possibly due to the oxidation of the copper on the surface of the gate metal. There was a significant difference in the surface color of the gate metal area of the device, which may indicate that the gate metal on the device surface had been oxidized and corroded.

**Figure 9:**
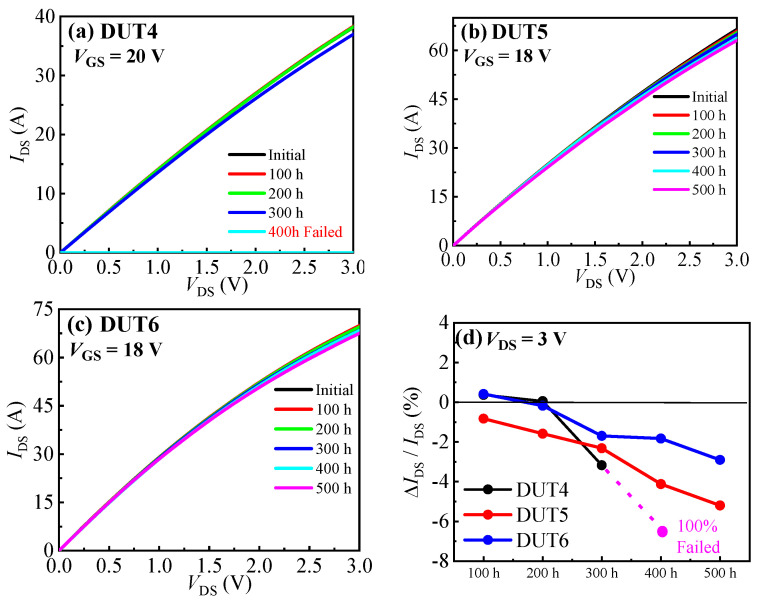
Output characteristic curves before and after UHAST of (**a**) DUT4, (**b**) DUT5, and (**c**) DUT6. (**d**) Summary of the change in the devices’ *I*_DS_.

**Figure 10:**
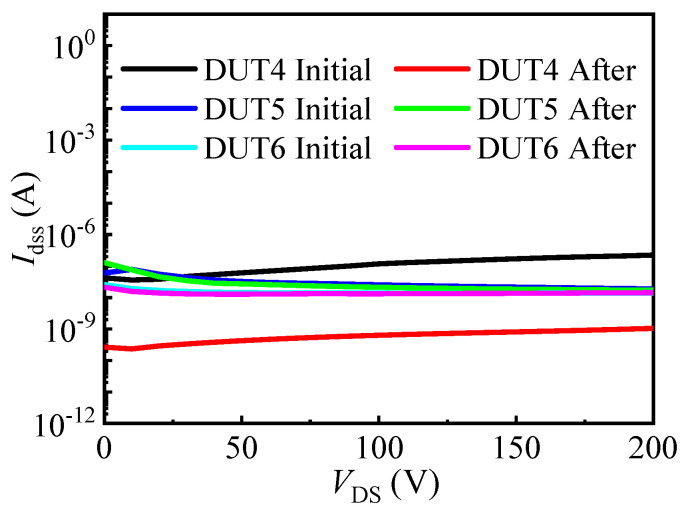
The drain leakage current characteristic curves before and after UHAST of the three types of SiC MOSFETs.

Figure 9 presents the output characteristic curves of the three types of SiC MOSFETs before and after the UHAST experiments. It can be observed that the conduction currents (@ V_GS_/V_DS_ = 18 V (20 V)/3 V) of all the SiC MOSFETs gradually decreased with the duration of the experiment. Before failure occurred in the planar gate structure SiC MOSFET, its conduction currents decreased by 3.17%. And after 500 h of UHAST experiments, the conduction currents of the other two structures of SiC MOSFETs decreased by 5.20% and 2.90%, respectively. As mentioned above, the presence of moisture in the UHAST experiment can lead to corrosion of the devices’ metal contacts and interconnects, which can increase the devices’ resistance, leading to the decrease in the devices’ conduction currents. Figure 10 displays the drain leakage current characteristics curves of the three types of SiC MOSFETs before and after the UHAST experiments. It can be observed that, after failure occurred in the planar gate structure SiC MOSFET, its drain leakage current decreased by more than two orders of magnitude. This further supports the possibility of a breakage in the drain or source electrode of the planar gate structure SiC MOSFET after 400 h of UHAST experiments. For the dual trench structure and asymmetric trench structure SiC MOSFETs, their drain leakage current remained largely unchanged after 500 h of UHAST experiments.

## 4. Conclusions

This work investigates the influences of hydrogen gas and high temperature, high humidity environments on the performance of SiC MOSFETs. The SiC MOSFETs exhibited a negative drift in threshold voltage, an increase in maximum transconductance, and an increase in drain current after the hydrogen effect experiment. And through long-term high temperature hydrogen effect experiments, it is found that high temperature does not accelerate the impact of hydrogen on the devices and electron mobility; instead, the prolonged exposure to high temperatures may increase the stress on the SiO_2_/SiC interface, leading to a decrease in electron mobility, subsequently reducing the transconductance and drain current. In the high temperature, high humidity experiment, it is found that high temperature, high humidity environments can cause a certain negative drift in the devices’ threshold voltage. Furthermore, the maximum transconductance and drain current gradually decreased as the duration of the experiments increased, which may because the presence of moisture can lead to corrosion of the devices’ metal contacts and interconnects.

## Figures and Tables

**Table 1:** Test settings for each DUT.

DUT1	DUT2	DUT3
C2M0080120D	SCT3040KL	IMW120R045M1
Hydrogen effect experiments		
DUT4	DUT5	DUT6
C2M0080120D	SCT3040KL	IMW120R045M1
High Accelerated Temperature and Humidity Stress Test		

## Data Availability

Data are available on request.
